# Evaluation of Pupillary Light Reflex in Amblyopic Eyes Using Dynamic Pupillometry

**DOI:** 10.4274/tjo.galenos.2019.32748

**Published:** 2019-12-31

**Authors:** Gülfidan Bitirgen, Mohammed Daraghma, Ahmet Özkağnıcı

**Affiliations:** 1 Necmettin Erbakan University Meram Faculty of Medicine, Department of Ophthalmology, Konya, Turkey; 2 Reyhanlı Sevgi Hospital, Clinic of Ophthalmology, Hatay, Turkey

**Keywords:** Amblyopia, anisometropia, dynamic pupillometry, pupillary light reflex, strabismus

## Abstract

**Objectives:**

To evaluate the pupillary light reflex responses in patients with unilateral strabismic and anisometropic amblyopia using dynamic pupillometry.

**Materials and Methods:**

A total of 102 eyes of 51 patients with unilateral amblyopia were included in this cross-sectional study. Of the 51 patients, 37 (72.5%) had strabismic amblyopia and 14 (27.5%) had anisometropic amblyopia. All patients underwent complete ophthalmological examination, and pupillary light reflex responses were measured using a computerized dynamic pupillometry system (MonPack One; Metrovision, France). Initial pupil diameter; the amplitude, latency, duration, and velocity of pupil contraction; and the latency, duration, and velocity of pupil dilation were recorded. Results obtained from the patients’ amblyopic and normal fellow eyes were compared using paired-samples t-test and Wilcoxon signed rank test.

**Results:**

The mean age of the patients was 11.9±6.0 years. Amblyopic eyes had longer contraction latency (p=0.009), shorter contraction duration (p=0.002), and higher dilation velocity (p=0.033) compared to fellow eyes, while other parameters did not show significant differences. In subgroup analysis, eyes with strabismic amblyopia had longer contraction latency (p=0.006) and shorter contraction duration (p=0.017), while eyes with anisometropic amblyopia had shorter contraction duration (p=0.030) when compared with fellow eyes.

**Conclusion:**

In this study, the objective records obtained by dynamic pupillometry showed that pupillary light reflex responses are affected in amblyopic eyes. This finding may shed light on unclear aspects of the pathophysiology of amblyopia.

## Introduction

Amblyopia is characterized by decreased visual acuity resulting from interrupted communication between the retina and visual cortex during development of the visual system, with no apparent organic pathology. Deprivation, strabismus, and refractive errors early in life lead to the loss of central visual functions such as visual acuity, contrast sensitivity, and visual field.^1^ With a reported prevalence of 2-4% in the population, amblyopia is the leading cause of preventable vision loss.^2,3,4^

Numerous studies have been conducted on the tissues affected in amblyopic eyes. Histopathological and clinical studies have revealed changes in the retina, optic nerve, lateral geniculate nucleus, and visual cortex in amblyopic eyes.^5,6,7^ Several studies have also examined the relationship between amblyopia and the pupillary light reflex, and reported changes in pupil diameter and the pupillary reflex in amblyopia.^8,9,10,11,12^ In addition, relative afferent pupillary defect in patients with unilateral amblyopia has been reported at rates ranging from 9% to 81.8%.^8,9,10^ It has been suggested that weak fixation and the projection of light stimuli extrafoveal retinal areas in amblyopic eyes may cause changes in pupillary reflexes.^10,11^

Minute pupillary changes may be undetectable in routine ophthalmologic examination. The introduction of automated infrared pupillometry devices has allowed the objective and quantitative measurement of pupil diameter and kinetic reflexes to light stimuli. Dynamic pupillometry has been widely used in recent years, especially for the evaluation of autonomic dysfunctions.^13,14,15^ Previous studies investigating pupillary changes in amblyopic patients have utilized methods such as neutral density filters, video pupillography, and wavefront analyzers. However, few studies have reported the results of dynamic infrared pupillometry. The aim of this study was to evaluate pupillary light reflex responses recorded with dynamic pupillometry in patients with unilateral strabismic and anisometropic amblyopia.

## Materials and Methods

The study included 102 eyes of 51 patients with amblyopia in one eye and no visual impairment in the fellow eye. Patients with visual acuity between 20/400 and 20/32 in the amblyopic eye or with at least two lines of difference between eyes and with visual acuity of 20/20 or better in the fellow eye were included in the study. Visual acuity was measured with a standard Snellen chart and converted to logarithm of the minimum angle of resolution (logMAR) units for statistical analysis. Anisometropia was defined as a spherical equivalent difference of at least 1.5 diopters between the eyes. Patients with deprivation amblyopia or organic eye disease, history of previous intraocular surgery, visual acuity worse than 20/400, and those who were unable to fixate or cooperate were excluded from the study. The study was approved by the Clinical Research Ethics Committee of the Necmettin Erbakan University (2018/1135). Informed consent forms were obtained from all patients in the study or their legal guardians.

**Figure 1:**
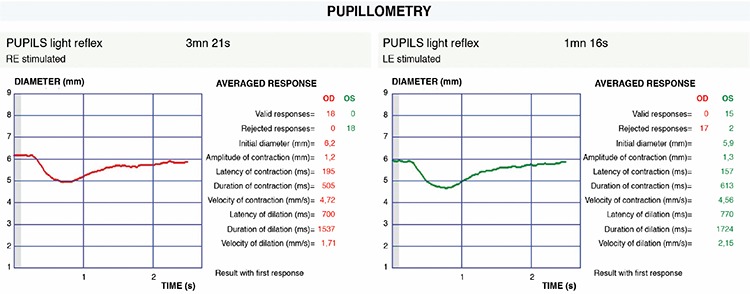
Pupillary light reflex responses recorded by dynamic pupillometry in a patient with strabismic amblyopia in the right eye

Each patient underwent detailed ophthalmologic examination including visual acuity measurement, cycloplegic refraction examination, strabismus examination, slit-lamp anterior segment examination, and dilated fundus examination. Pupillary light reflex responses were evaluated with dynamic pupillometry (MonPackOne®; Metrovision, France). The device is equipped with infrared illumination (880 nm) and a high-resolution infrared sensor that allows measurement of pupil parameters in complete darkness. Pupillary responses were elicited with white light stimulus in a completely dark environment (light intensity: 100 cd/m^2^, on/off duration: 200/3300 ms) and recorded with measurement sensitivity of 0.1 mm. The patient was allowed 5 minutes for dark adaptation and the measurements of both eyes were performed monocularly. Initial pupil diameter; the amplitude, latency, duration, and velocity of pupil contraction; and the latency, duration, and velocity of pupil dilation were measured from the patients’ amblyopic and healthy eyes and compared (Figure 1).

### Statistical Analyses

SPSS version 17.0 software package was used for statistical analyses of the data (SPSS for Windows, Chicago, USA). The Shapiro–Wilk test was used to evaluate conformity of quantitative data to normal distribution. When comparing data obtained from the amblyopic eyes and healthy eyes of the patients, a paired-samples t-test was used for normally distributed data, and Wilcoxon signed rank test was used for non-normally distributed data. Pearson and Spearman correlation tests were used to analyze correlation between pupillary responses and depth of amblyopia. Differences with p values less than 0.05 were considered statistically significant.

## Results

Thirty-seven (72.5%) patients in the study had strabismic amblyopia and 14 (27.5%) had hypermetropic anisometropic amblyopia. Of the patients with strabismic amblyopia, 29 (78.4%) had esotropia and 8 (21.6%) had exotropia. The mean age of the patients was 11.9±6.0 years. Snellen visual acuity in the amblyopic eyes ranged from 20/400 to 20/32 (mean 0.56±0.30 logMAR). Visual acuity was 20/20 or better in all fellow eyes.

Compared to the healthy fellow eyes, amblyopic eyes exhibited longer pupil contraction latency (median 205.0 vs. 246.0 ms, respectively; p=0.009), shorter contraction duration (median 613.0 vs. 562.0 ms, respectively; p=0.002), and higher dilation velocity (median 2.18 vs. 2.23 mm/s, respectively; p=0.033). There were no significant differences in other parameters between the two eyes (Table 1). Subgroup analysis showed that in patients with strabismic amblyopia, amblyopic eyes had longer contraction latency (median 252.0 vs. 193.0 ms; p=0.006) and shorter contraction duration (median 566.0 vs. 613.0 ms; p=0.017) compared to the healthy eyes, with no significant differences in terms of other parameters (Table 2). In patients with anisometropic amblyopia, contraction duration was shorter in amblyopic eyes compared to healthy eyes (median 550.0 vs. 613.0 ms, respectively; p=0.030), with no significant differences in the other parameters (Table 3).

Correlation analyses did not reveal any significant relationship between the visual acuity and pupillary light reflex parameters of amblyopic eyes.

## Discussion

Although amblyopia is defined as a decrease in visual acuity without structural damage to the eye and visual pathways, cell shrinkage has been demonstrated in the parvocellular layers of the lateral geniculate nucleus.^5,16^ Changes in the macula, peripapillary retinal nerve fiber layer, and choroidal thickness have also been reported in optical coherence tomography studies.^17,18,19^ Longer latencies and lower amplitudes in visual-evoked potential (VEP) and markedly reduced responses in pattern electroretinography have been documented in electrophysiological tests of amblyopic eyes.^20,21^

Considering the possibility that retinal ganglion cells and anterior visual pathways may be affected in amblyopic eyes, studies have also been conducted to evaluate pupillary light reflexes. Measurements with neutral density filters have revealed relative afferent pupillary defect in amblyopic eyes.^9,10^ Portnoy et al.^10^ demonstrated the presence of afferent pupillary defect in 45 of 55 amblyopic patients using this method and suggested that this defect may be due to poor fixation ability and incomplete development of the foveal ganglion cells in amblyopic eyes. In a study based on infrared pupillography, patients with strabismic and anisometropic amblyopia exhibited prolonged pupil contraction latency in the direct light reflex response, but no prolonged latencies were observed in indirect responses.^22^ The authors therefore proposed that afferent fibers were responsible for prolonged latency and that there was no pathology in the pupillomotor efferent system. In the same study, examination of 8 amblyopic patients who improved with treatment revealed no prolongation of pupil contraction latency, suggesting that latency prolongation may be a reversible phenomenon that can resolve with amblyopia treatment. In a study evaluating the multifocal VEP test results of amblyopic patients, it has been reported that VEP latency was longer and amplitude was lower in amblyopic eyes.^20^ Our findings that amblyopic eyes had prolonged pupil contraction latency, or in other words, showed a longer delay between light stimulus and pupil contraction compared to healthy fellow eyes supports the view that afferent transmission is slowed in amblyopia.

Dynamic pupillometry enables the objective and reliable recording of pupillary light reflex responses and independent evaluation of several parameters that reflect pupil movements. In their study of the dynamic pupillometry responses of patients with hypermetropic anisometropic amblyopia, Yetkin et al.^23^ reported that the pupil contraction amplitude of amblyopic patients was lower compared to that of healthy subjects, while no difference was detected in other parameters. In the present study, we observed longer pupil contraction latency, shorter contraction duration, and greater dilation velocity in patients’ amblyopic eyes compared to their healthy fellow eyes, with no significant differences in terms of initial pupil diameter or other parameters. Yuksel et al.^24^ evaluated the anterior segment parameters and pupil diameters of patients with hypermetropic anisometropic amblyopia using the Pentacam device and reported no difference in pupil diameter between amblyopic eyes and normal eyes. In their study evaluating the pupil diameters of patients with anisometropic amblyopia under mesopic conditions using an ocular wavefront analyzer, Kocamış et al.^12^ reported smaller pupil diameter in amblyopic eyes. It is known that differences in refractive error can also affect pupil diameter. Another study using a wavefront analyzer on non-amblyopic patients showed that refractive error was correlated with the mesopic pupillary diameter, with larger pupil diameters in myopic patients.^25^ In that study, initial pupil diameters did not differ significantly between amblyopic and healthy eyes both in patients with strabismic amblyopia and those with hypermetropic anisometropic amblyopia. In our study, initial pupil diameter measurements were obtained by the dynamic pupillometry device under scotopic conditions. Inconsistent results reported in the literature on this issue may be attributable to differences in the lighting conditions under which measurements are made and the sensitivities of the devices used. In addition, the limited number of patients with anisometropic amblyopia in our study, which is one of its limitations, may have contributed to the lack of statistical significance.

Portnoy et al.^10^ reported that there was no correlation between the degree of relative afferent pupil defect detected in amblyopic eyes and the type or depth of amblyopia. Other studies evaluating the correlation between visual acuity level and pupil diameter and light reflexes in amblyopic eyes also failed to reveal a significant relationship.^12,22^ Similarly, none of the pupillary light reflex parameters examined in the present study showed significant correlation with depth of amblyopia in our patients.

One of the limitations of this study is that its cross-sectional design precludes an evaluation of whether pupillary light reflex responses changed in patients whose visual acuity in the amblyopic eye improved with treatment. The small number of patients with anisometropic amblyopia and the absence of patients with myopic anisometropia are other limitations of the study.

## Conclusion

In this study, the pupils of amblyopic eyes were found to contract later in response to light, remain contracted for a shorter time, and dilate faster. These findings may not only facilitate early diagnosis of amblyopia, but may also shed light on the unexplained mechanisms involved in its pathophysiology. Long-term studies of amblyopic patients will also allow investigation into whether improved visual acuity after treatment is associated with changes in pupil responses.

## Figures and Tables

**Table 1:**
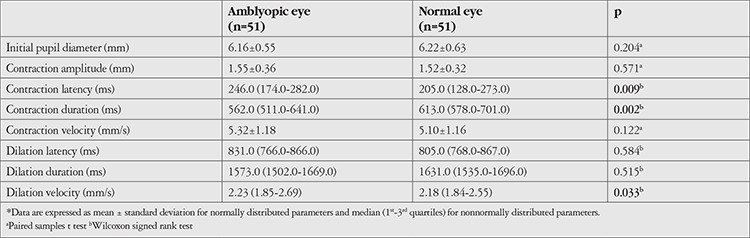
Pupillary light reflex responses of the patients’ amblyopic eyes and normal fellow eyes

**Table 2:**
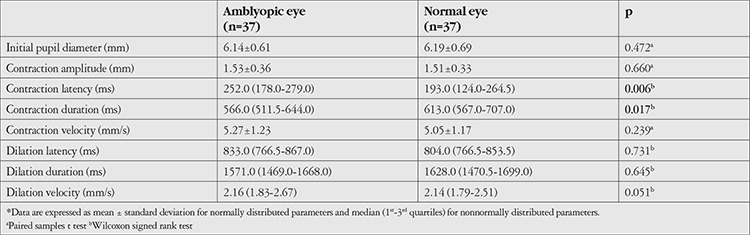
Pupillary light reflex responses in patients with strabismic amblyopia

**Table 3:**
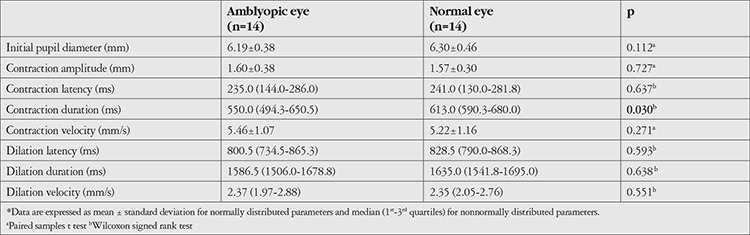
Pupillary light reflex responses in patients with anisometropic amblyopia
